# Laparoscopic Ladd procedure in infants: Report of three cases from a developing country

**DOI:** 10.4103/0972-9941.43093

**Published:** 2008

**Authors:** Mohammed A. Youssef

**Affiliations:** Department of Paediatric Surgery, Alexandria Hospital for Sick Children, Health Insurance Authority, Alexandria, Egypt

**Keywords:** Laparoscopic Laddaes procedure, Malrotation, Volvulus

## Abstract

Infants with intestinal malrotation present with bilious emesis and the diagnosis is generally obtained by an upper gastrointestinal barium study. Malrotation is suspected if the ligament of Treitz is not positioned to the left of the vertebral body. Three patients were admitted to our department from March 2006 to May 2007, aged three weeks, one month and eight months, weighing 3,3.200 and 8 kg respectively to whom laparoscopic Ladd's procedure was done successfully.

## INTRODUCTION

Infants with intestinal malrotation present with bilious emesis and the diagnosis is generally obtained by an upper gastrointestinal barium study. Malrotation is suspected if the ligament of Treitz is not positioned to the left of the vertebral body. Barium enema may also be used to detect malrotation by noting the abnormal position of the cecum from its usual placement in the right lower quadrant, but this study is not as reliable due to the mobility of the cecum. Some infants may not have classic radiographic findings for malrotation, yet the contrast studies are not entirely normal.

## MATERIALS AND METHODS

Three patients were admitted to our department from March 2006 to May 2007, aged three weeks, one month and eight months, weighing 3,3.200 and 8 kg respectively.

All patients had symptoms of intermittent upper intestinal obstruction, and malrotation was documented by an upper gastrointestinal contrast study. None of the patients had acute volvulus. The procedure was done in all the cases under general anaesthesia, using 5 mm diameter telescope and 3 mm instruments. Port of telescope was placed in the infraumbilical ring, and the instruments right and left mid to lower quadrants without ports. A standard Ladd's procedure with division of the Ladd's bands, incision of the common mesentery and appendectomy. The jejunum and ileum were positioned on the right and the colon on the left in the abdominal cavity.

Laparoscopic Ladd's procedure was done successfully in the three cases. Operative times averaged 80 min (58,62,120 minutes respectively). Feedings were started on the first postoperative day in two cases and on the second postoperative day in two cases. Hospital stay ranged from two to four days (average, 2.2). There were no complications. All patients had resolution of their symptoms.

## DISCUSSION

Neonates with a short history of bilious vomiting are most likely to have MGV-complicating malrotation, but older children who have chronic intermittent symptoms are also at risk. Since there is no way to predict which patients will develop catastrophic bowel necrosis, early diagnosis and operation are necessary to prevent mortality and short-gut syndrome.[1]

Children with malrotation who are older than two years old have a significant risk of volvulus that is difficult to predict radiologically. They require surgical attention even if asymptomatic. Laparoscopy allows evaluation of the base of the mesentery and completion of the Ladd's procedure.[2]

The treatment of the intestinal malrotation with or without midgut volvulus with the Ladd procedure for laparoscopic way has been proposed by several authors since 1995.[3]

Laparoscopic Ladd's procedure is a safe and effective technique. It can be performed in neonates in times equivalent to standard open techniques, and it appears to allow for earlier feeds and decreased hospital stays allows a good visualisation of this congenital abnormality, and it is easy to perform with a significantly reduced operative trauma.[4,5]

## CONCLUSION

Laparoscopic Ladd's procedure is a safe and effective technique for children and infants.
